# Inflammation and Dysregulated Bone Turnover Confound Serum ICAM-1 as a Cardiovascular Marker in Hemodialysis

**DOI:** 10.3390/biom16010102

**Published:** 2026-01-07

**Authors:** Maria Divani, Aikaterini Katsanaki, Panagiota Makri, Christina Poulianiti, Evangelos Lykotsetas, Andriani Balatsouka, Maria Tziastoudi, Ioannis Stefanidis, Theodoros Eleftheriadis

**Affiliations:** Department of Nephrology, Faculty of Medicine, University of Thessaly, Biopolis, Mezourlo Hill, 41110 Larissa, Greece; madivani@med.uth.gr (M.D.); akatsanaki@gmail.com (A.K.); pmakri04@gmail.com (P.M.); christinepoulianiti@gmail.com (C.P.); evanlyko@gmail.com (E.L.); andrianna.bal@gmail.com (A.B.); matziast@med.uth.gr (M.T.); stefanid@med.uth.gr (I.S.)

**Keywords:** ICAM-1, inflammation, bone metabolism, cardiovascular disease, hemodialysis

## Abstract

Cardiovascular disease (CVD) remains the leading cause of mortality among hemodialysis (HD) patients, underscoring the need for reliable biomarkers for early diagnosis and management. Serum intercellular adhesion molecule-1 (ICAM-1) has been investigated for years as a potential CVD marker but has yet to establish clinical utility. In a cohort of 142 HD patients, we examined the potential of serum ICAM-1 as a CVD biomarker and evaluated whether confounding factors, including low-grade inflammation and chronic kidney disease–mineral bone disorder (CKD-MBD), limit its diagnostic value. In addition to serum ICAM-1, routine biochemical parameters, bone alkaline phosphatase (bALP), and nitric oxide (NO) were measured. Serum levels of ICAM-1, bALP, and NO did not differ between patients with and without CVD, defined by a positive history of coronary heart disease, stroke, or peripheral arterial disease. Serum ICAM-1 concentrations were higher in HD patients with inflammation, as indicated by C-reactive protein levels >1 mg/dL. ICAM-1 showed no correlation with NO, a marker of endothelial dysfunction, but was positively correlated with bALP, a marker of CKD-MBD. In conclusion, serum ICAM-1 is not a reliable biomarker of CVD in HD patients. Its diagnostic utility appears confounded by inflammation and disturbances in bone turnover.

## 1. Introduction

Cardiovascular disease (CVD) represents the leading cause of mortality among patients with end-stage kidney disease (ESKD) [1]. This association is particularly pronounced in younger individuals. For example, the annual cardiovascular mortality rate among patients aged 25 to 34 years with ESKD is approximately 500 to 1000 times higher than that observed in age-matched individuals from the general population [2].

Therefore, identifying reliable markers of CVD in this population is of substantial importance, as it would facilitate earlier, more targeted diagnostic evaluations and, when indicated, timely therapeutic interventions. One such marker is intercellular adhesion molecule-1 (ICAM-1), which is regarded as an indicator of endothelial activation [3]. In the general population, elevated ICAM-1 levels have been associated with an increased risk of cardiovascular disease [3,4,5,6,7]. Additionally, endothelial dysfunction and activation are associated with the burden of established atherosclerotic disease. In 89 consecutive patients undergoing coronary angiography, flow-mediated dilation at the brachial artery was a reliable indicator of coronary artery disease burden [8]. Another study showed that in patients with coronary heart disease, VCAM-1 level is an indicator of atherosclerotic plaque instability and is related to the possibility of acute coronary syndrome development [9]. The same relation was detected for serum ICAM-1 levels. Elevation of ICAM-1 levels in coronary heart disease patients was associated with increased risk of secondary coronary events independently of other traditional risk factors [10]. In patients undergoing hemodialysis (HD), serum ICAM-1 levels are elevated and are associated with malnutrition [11], progression of vascular calcification [12], sub-clinical atherosclerosis, assessed by increased carotid intima-media thickness, and future cardiovascular mortality [13], and overall risk for cardiovascular mortality [14]. Serum ICAM-1 levels have also been associated with established CVD. In a cohort of 106 HD patients, serum ICAM-1 levels were higher in 36 patients with peripheral arterial disease [15]. In another cohort of 81 HD patients, serum ICAM-1 levels were higher in 25 patients with CVD. Seventeen of them had a history of myocardial infarction, coronary artery bypass, or signs of angina pectoris, 3 had a history of stroke, and 5 had peripheral arterial disease [16]. In a small cohort of HD patients with diabetes mellitus, 17 of 26 had CVD, defined as coronary heart disease, peripheral arterial disease, or cerebrovascular disease. Serum ICAM-1 was elevated in those with CVD [17]. Finally, in a cohort of 87 patients with ESKD, serum ICAM-1 levels were elevated in the 26 with CVD, defined as coronary heart disease, peripheral arterial disease, or cerebrovascular disease [18].

In addition to increased production, high serum levels of ICAM-1 in HD patients have been suggested to result from reduced renal clearance. Indeed, in one study, serum ICAM-1 levels increased with CKD stage [19]. On the other hand, other studies failed to detect an association between creatinine clearance or e-GFR and serum ICAM-1 levels [20,21]. Given the molecular weight of ICAM-1, which ranges from 60 to 114 kDa depending on its glycosylation state [22], and the glomerular filtration barrier [23], renal clearance of serum ICAM-1 is likely not significant.

Despite numerous publications over the past decades, serum ICAM-1 has not been established in clinical practice as a reliable marker of CVD in HD patients. One potential explanation is publication bias, whereby studies reporting negative or non-significant results may have remained unpublished. Confounding factors prevalent in HD patients may influence serum ICAM-1 levels, limiting its utility as a marker of CVD. One such factor is the chronic low-grade inflammation characteristic of this population [24]. Indeed, a positive correlation has been observed between serum ICAM-1 levels and C-reactive protein (CRP) [16,25].

Another potential confounding factor is chronic kidney disease–mineral bone disorder (CKD-MBD), a condition commonly observed in patients undergoing HD [26]. In a cohort of 73 HD patients, serum ICAM-1 levels showed no association with carotid intima-media thickness or CRP but were correlated with serum calcium and phosphorus levels [27]. Interestingly, in patients with CKD stages 3–4, cholecalciferol administration led to a reduction in intact parathyroid hormone (iPTH) and ICAM-1 levels, along with an improvement in brachial artery flow-mediated dilation [28], and in HD patients, the same treatment decreased serum ICAM-1 [29]. Additionally, in HD patients with secondary hyperparathyroidism, treatment with cinacalcet alone (without vitamin D receptor agonists) led to reductions in iPTH, calcium, and phosphate levels, improvement in brachial artery flow-mediated dilation, and a decrease in serum ICAM-1, without affecting CRP levels [30].

Elevated serum ICAM-1 concentrations are typically indicative of endothelial activation, a condition characterized by enhanced expression of adhesion molecules that facilitate leukocyte recruitment and contribute to the development of atherosclerotic plaques [3]. However, endothelial dysfunction is also a defining feature in patients undergoing HD [31]. Traditionally, the term “endothelial dysfunction” refers to impaired vasodilation or disturbances affecting the endothelium’s vasoprotective homeostatic functions, with reduced nitric oxide (NO) bioavailability playing a central role in its pathogenesis [32]. Interestingly, a pioneering study demonstrated that NO suppresses endothelial activation by downregulating the expression of adhesion molecules and inhibiting the production of proinflammatory cytokines [33]. Thus, if serum ICAM-1 levels accurately reflect the state of the arterial endothelium, they would be expected to correlate negatively with serum NO levels.

In this study, we investigated whether serum ICAM-1 could serve as a biomarker for CVD, defined as a medical history of coronary heart disease, stroke, or peripheral arterial disease in HD patients. We examined the influence of low-grade inflammation, assessed by CRP, on serum ICAM-1 levels. Additionally, we evaluated the impact of bone turnover on serum ICAM-1, using serum bone alkaline phosphatase (bALP) as a marker. Serum bALP is recommended for assessing bone metabolism in HD patients according to KDIGO guidelines [26]. As bALP is produced by osteoblasts, it directly reflects bone turnover and may be a more reliable indicator than intact iPTH, which correlates poorly with bone biopsy results [34]. Finally, we assessed the relationship between endothelial dysfunction, as determined by serum NO levels, and serum ICAM-1.

## 2. Materials and Methods

### 2.1. Patients

A total of 142 stable HD patients were enrolled in this cross-sectional study, with a mean age of 66.36 ± 11.74 years; 102 were male, and 40 were female. The primary causes of ESKD were diabetic nephropathy in 37 patients, primary glomerulonephritis in 28, hypertension in 23, autosomal dominant polycystic kidney disease in 12, cardiorenal syndrome in 8, secondary focal segmental glomerulosclerosis in 6, vasculitis in 4, obstructive nephropathy in 3, analgesic nephropathy in 1, and unknown causes in 20 patients. Fifty patients had diabetes mellitus, and 52 had CVD, defined as a history of coronary heart disease (44 patients), stroke (12 patients), or peripheral arterial disease (14 patients). Notably, among the 12 patients with stroke, 10 also had coronary heart disease. Additionally, of the 14 patients with peripheral arterial disease, 8 also had coronary heart disease.

Patients underwent regular HD sessions using polysulfone dialyzers and a bicarbonate-based dialysate containing calcium at either 1.25 or 1.5 mmol/L. Each HD session lasted four hours and was conducted three times per week. The use of the phosphate binder sevelamer hydrochloride, the vitamin D analog paricalcitol, and the calcimimetic etelcalcetide was determined at the discretion of the treating nephrologists to achieve the KDIGO-recommended target levels for serum PTH, calcium, and phosphorus [26]. Ninety-four patients were taking statin therapy, and most of them were taking antihypertensives.

An inclusion criterion for the study was to be under HD treatment for at least six months prior to the study. Exclusion criteria were active infections, autoimmune diseases, malignancies, and liver pathologies. Also, patients who had received cytotoxic or immunosuppressive medications, corticosteroids, or bisphosphonates within six months prior to the study were excluded. Furthermore, 10 patients receiving nitrate therapy were excluded from the analysis of serum NO levels.

Twenty-six healthy individuals (mean age 64.35 ± 6.68 years; 18 males and 8 females) served as the control group after undergoing a review of their medical history and a physical examination.

Informed consent was obtained from all participants enrolled in the study. The study protocol was approved by the Ethics Committee of the Faculty of Medicine, University of Thessaly, Larissa, Greece (approval no. 558/10-2-2017).

### 2.2. Methods

Data were gathered from two hemodialysis units: one at the University Hospital of Larissa, Larissa, Greece and the other at the Chronic Hemodialysis Unit Eftichios Patsidis, Larissa, Greece. Clinical data on age, gender, duration of HD treatment, residual diuresis, hypertension, diabetes mellitus, CVD, coronary heart disease, stroke, peripheral artery disease, body mass index, and use of antihypertensive medications, nitrates, statins, antiplatelet medications, phosphate binders, vitamin D analogs, and calcimimetics were recorded. Additionally, the following routine laboratory data were assessed: white blood cell count, neutrophils, lymphocytes, hemoglobin, platelets, urea, creatinine, urea reduction ratio, cholesterol, triglycerides, LDL cholesterol, albumin, alkaline phosphatase, ferritin, transferrin saturation, calcium, phosphorus, intact parathyroid hormone, and C-reactive protein.

Blood samples were collected at the start of the second dialysis session of the week, and the serum was stored at –80 °C.

Serum ICAM-1 levels were measured using the EliKine Human CD54 ELISA Kit (Abkine, Atlanta, GA, USA), with a sensitivity of 7.8 pg/mL. Serum bALP was determined using the Human BALP ELISA Kit (FineTest, Wuhan, Hubei, China), which has a sensitivity of 1.875 ng/mL. Finally, serum NO concentrations were measured colorimetrically using an improved Griess method with the CheKine Micro Nitric Oxide (NO) Assay Kit (Abkine), which measures both nitrate and nitrite. All other evaluated parameters were measured and recorded as part of routine laboratory assessments, performed at the same time as serum collection for the measurements of the factors described above.

### 2.3. Statistical Analysis

Statistical analyses and graphical representations were conducted using IBM SPSS Statistics version 29 (IBM Corp., Armonk, NY, USA). The normality of the evaluated variables was assessed using the one-sample Kolmogorov–Smirnov test. Because ICAM-1, BALP, and NO did not follow a normal distribution, nonparametric tests were applied. Group comparisons were performed using the Mann–Whitney U test, and results were presented as median (interquartile range). Correlations were evaluated using Spearman’s rho. Linear regression analysis was also performed after verifying the assumptions of linearity, normality of residuals, homoscedasticity, and absence of multicollinearity. A *p*-value of <0.05 was considered statistically significant.

## 3. Results

### 3.1. Patients’ Clinical and Laboratory Data

The clinical and laboratory data of the patients evaluated are presented in Table 1.

### 3.2. Serum ICAM-1, bALP, and NO Levels in HD Patients and Healthy Subjects

**Figure 1:**
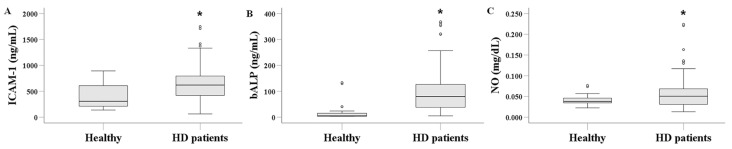
Comparison of serum ICAM-1 (**A**), bALP (**B**), and NO (**C**) between healthy subjects and HD patients. Serum levels of ICAM-1, bALP, and NO were significantly higher in HD patients compared to healthy individuals. The line within the box indicates the median value. The box represents the interquartile range (IQR), while the whiskers show the data range, extending 1.5 × IQR above the third quartile and below the first quartile. Dots beyond the whiskers denote outliers. An asterisk indicates a *p*-value less than 0.05.

Serum ICAM-1 levels were significantly higher in HD patients, measuring 619.853 (421.250–794.944) ng/mL, compared to 307.581 (208.380–615.913) ng/mL in healthy subjects (*p* < 0.001) (Figure 1A). Similarly, serum bALP levels were elevated in HD patients at 79.368 (38.075–126.538) ng/mL, versus 5.236 (3.567–17.181) ng/mL in healthy controls (*p* < 0.001) (Figure 1B). Finally, serum NO levels were 0.051 (0.031–0.069) mg/dL in HD patients and 0.038 (0.033–0.046) mg/dL in healthy subjects (*p* = 0.041) (Figure 1C).

### 3.3. Serum ICAM-1, bALP, and NO in HD Patients with or Without CVD

**Figure 2:**
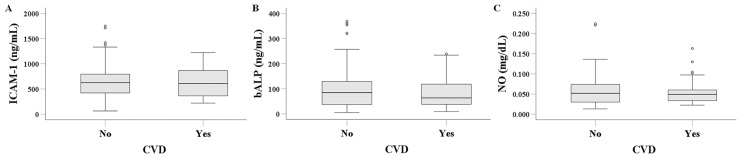
Comparison of serum ICAM-1 (**A**), bALP (**B**), and NO (**C**) between HD patients with or without CVD. Serum levels of ICAM-1, bALP, and NO did not differ significantly between HD patients with CVD and those without CVD.

Serum ICAM-1 levels were 627.602 (361.493–865.639) ng/mL in HD patients with CVD and 625.096 (422.539–793.742) ng/mL in those without CVD (*p* = 0.919) (Figure 2A). Similarly, serum bALP levels did not differ significantly between the two groups, measuring 64.167 (38.075–135.800) ng/mL in patients with CVD and 84.486 (35.872–129.604) ng/mL in patients without CVD (*p* = 0.391) (Figure 2B). Finally, serum NO levels were 0.049 (0.033–0.059) mg/dL in HD patients with CVD and 0.052 (0.030–0.074) mg/dL in those without CVD (*p* = 0.673) (Figure 2C).

### 3.4. Serum ICAM-1, bALP, and NO in HD Patients with or Without Diabetes Mellitus

**Figure 3:**
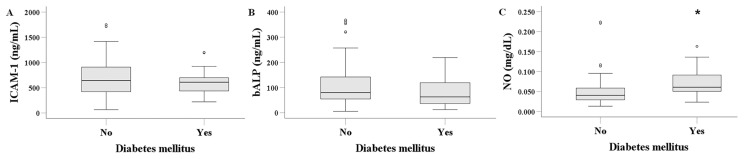
Comparison of serum ICAM-1 (**A**), bALP (**B**), and NO (**C**) between HD patients with or without diabetes mellitus. Serum levels of ICAM-1 and bALP showed no significant difference between HD patients with diabetes mellitus and those without diabetes mellitus. Serum NO levels were higher in HD patients with diabetes mellitus. An asterisk indicates a *p*-value less than 0.05.

Serum ICAM-1 levels did not differ significantly between HD patients with and without diabetes mellitus. Median levels were 608.834 (420.809–700.347) ng/mL in patients with diabetes mellitus and 640.794 (420.290–914.894) ng/mL in those without (*p* = 0.088) (Figure 3A). Similarly, serum bALP levels showed no significant difference, measuring 62.825 (33.913–120.597) ng/mL in patients with diabetes mellitus and 79.728 (52.460–142.187) ng/mL in those without (*p* = 0.104) (Figure 3B). In contrast, serum NO levels were significantly higher in HD patients with diabetes mellitus, at 0.061 (0.051–0.092) mg/dL, compared to 0.041 (0.029–0.059) mg/dL in patients without diabetes mellitus (*p* < 0.001) (Figure 3C).

### 3.5. Serum ICAM-1, bALP, and NO in HD Patients with or Without Low-Grade Inflammation

A CRP level above 1 mg/dL was used to define dialysis-related low-grade inflammation. Interestingly, 104 patients exhibited CRP levels above 1 mg/dL, with no significant difference observed between HD patients with and without CVD. The median CRP level was 1.470 (1.028–1.820) mg/dL in patients without CVD and 1.170 (0.915–1.800) mg/dL in those with CVD (*p* = 0.230).

**Figure 4:**
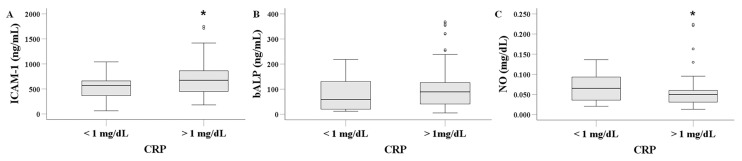
Comparison of serum ICAM-1 (**A**), bALP (**B**), and NO (**C**) between HD patients with or without inflammation. Defining inflammation as a CRP level > 1 mg/dL, HD patients with inflammation had higher serum ICAM-1 levels than those without inflammation. Serum bALP did not differ between patients with or without inflammation. HD patients with inflammation had lower serum NO levels than those without inflammation. An asterisk indicates a *p*-value less than 0.05.

Serum ICAM-1 levels were significantly higher in HD patients with inflammation than in those without, at 671.560 (448.426–868.776) ng/mL versus 568.013 (360.882–660.318) ng/mL, respectively (*p* = 0.009) (Figure 4A). In contrast, serum bALP levels did not differ significantly between the two groups, with values of 89.321 (42.597–126.378) ng/mL in patients with inflammation and 58.376 (20.113–131.203) ng/mL in those without (*p* = 0.209) (Figure 4B). Finally, serum NO levels were lower in HD patients with inflammation (0.049 [0.031–0.060] mg/dL) than in those without inflammation (0.065 [0.036–0.094] mg/dL; *p* = 0.047) (Figure 4C).

### 3.6. Correlations Among ICAM-1, bALP, NO, and Other Factors

In HD patients, serum ICAM-1 showed a positive correlation with bALP (Rho = 0.204, *p* = 0.016), but no significant correlation with NO (Rho = −0.121, *p* = 0.165). Additionally, NO was not correlated with bALP (Rho = −0.061, *p* = 0.492) (Table 2).

Using the bALP median as a cutoff, ICAM-1 levels were higher in patients with bALP above the median (697.818 (399.884) ng/mL vs. 572.981 (352.410) ng/mL, *p* = 0.002).

Among the other factors listed in Table 1, serum ICAM-1 was significantly correlated only with duration of HD treatment (Rho = 0.186, *p* = 0.026) and ALP (Rho = 0.239, *p* = 0.004). Notably, a multiple linear regression analysis predicting serum ICAM-1 from bALP and ALP was significant (R = 0.399, *p* < 0.001), with both bALP (standardized b = 0.232, *p* = 0.004) and ALP (standardized b = 0.286, *p* < 0.004) serving as positive predictors. A further multiple linear regression, including all factors related to serum ICMA-1 levels in this study, i.e., bALP, ALP, inflammatory status (defined by a CRP cut-off of 1 mg/dL), and duration on HD treatment, demonstrated that bALP, ALP, and inflammatory status independently and positively predicted serum ICAM-1 levels. In contrast, duration of HD treatment was not a significant predictor (Table 3).

Regarding the additional factors presented in Table 1, bALP showed significant correlations with age (Rho = −0.271, *p* = 0.002), duration on HD treatment (Rho = 0.261, *p* = 0.002), urea (Rho = 0.263, *p* = 0.002), creatinine (Rho = 0.266, *p* = 0.001), CRP (Rho = 0.177, *p* = 0.036), ALP (Rho = 0.312, *p* < 0.001), calcium (Rho = 0.168, *p* = 0.047), phosphorous (Rho = 0.186, *p* = 0.028), and iPTH (Rho = 0.594, *p* < 0.001).

Finally, regarding the remaining factors shown in Table 1, NO was significantly correlated with WBC (Rho = 0.173, *p* = 0.047), triglycerides (Rho = 0.282, *p* = 0.001), LDL (Rho = −0.283, *p* < 0.001), and phosphorus (Rho = 0.179, *p* = 0.040).

Notably, we also calculated another inflammation marker, the neutrophil-to-lymphocyte ratio (NLR). However, no correlation was found between NLR and CRP (Rho = 0.029, *p* = 0.730), ICAM-1 (Rho = −0.118, *p* = 0.162), NO (Rho = −0.052, *p* = 0.557), or bALP (Rho = −0.073, *p* = 0.389). The lack of correlation between NLR and CRP seems unusual, but it has also been observed in other studies [35,36,37]. Therefore, we used CRP in our analysis as a more established marker of inflammation.

In healthy subjects, serum ICAM-1 showed no significant correlation with either bALP (Rho = −0.330, *p* = 0.115) or NO (Rho = −0.347, *p* = 0.097). Additionally, NO was not correlated with bALP (Rho = −0.234, *p* = 0.250).

## 4. Discussion

Given that CVD represents the leading cause of mortality among HD patients [1,2], the identification of potential biomarkers for the early detection of CVD in this population is of critical importance. One such potential biomarker is serum ICAM-1. ICAM-1 is widely recognized as an indicator of endothelial activation [3]. Studies conducted in both the general population and among HD patients have yielded encouraging results, suggesting its potential utility as a marker of CVD [3,4,5,6,7,11,12,13,14,15,16]. However, despite extensive research over several years, serum ICAM-1 has not yet been established as a clinically applicable biomarker for CVD in HD patients. The presence of confounding factors and comorbid conditions—such as low-grade inflammation, CKD-MBD, and diabetes mellitus—commonly observed among HD patients [24,26,38], may contribute to this limitation.

We observed that serum ICAM-1 levels were elevated in HD patients compared to healthy controls. This finding aligns with previous studies and, according to the mainstream view, reflects endothelial activation in this population [3,11,12,13,14,15,16]. We also found that bALP, a marker of bone turnover, was elevated in HD patients. This finding is expected, given the presence of CKD-MBD that characterizes this population [26]. Interestingly, we observed a positive correlation between bALP and other serum markers of bone turnover, including iPTH, calcium, phosphorus, and total ALP. Compared with healthy controls, serum NO levels were elevated in HD patients, as well. This finding appears to contradict the well-established observation that endothelial dysfunction is common among HD patients [31], with reduced nitric NO bioavailability being one of its primary underlying causes [32]. However, elevated serum NO levels in HD patients have also been reported in previous studies. These findings have been attributed either to reduced NO clearance during dialysis or to increased NO synthesis in response to uremic toxins. In the latter case, it has been suggested that elevated NO production may represent a compensatory defense mechanism against uremia and the associated hypertension [39,40]. It should be noted that because NO is extremely short-lived (half-life in seconds), it is usually measured indirectly by analyzing its downstream products, nitrite and nitrate. Therefore, it cannot detect rapid fluctuations in NO levels.

Defining CVD as a documented history of coronary heart disease, stroke, or peripheral arterial disease, we did not observe a significant difference in serum ICAM-1 levels between HD patients with or without CVD. This finding is inconsistent with previous studies that reported an association between serum ICAM-1 levels and atherosclerosis in HD patients [11,12,13,14,15,16,17,18]. Regarding studies that evaluated the association between serum ICAM-1 and established CVD, differences among patients may account for the divergent findings. In Cheng et al.’s cohort, in addition to the racial difference from our study, the percentage of patients with peripheral arterial disease was extremely high, at 34% [15]. The study by Moldovan et al. was relatively small (*n* = 26 patients), included only diabetic patients, and most of them (n = 15) had CVD [17]. The study by Stenvinkel et al. included ESKD patients who were not yet on HD at the start of the study, when higher serum ICAM-1 levels were observed in those with established CVD [18]. Finally, in Papayanni’s study, the HD patients were somewhat younger, 40% were dialyzed with less biocompatible modified cellulose membranes, and importantly, patients with diabetes mellitus were excluded [16]. Interestingly, parameters related to CKD-MBD were not evaluated in these studies. In addition, the fact that despite decades of research, serum ICAM-1 has not been established as a reliable CVD marker in HD patients, could be the result of publication bias and confounding factors common in this population. Similarly to serum ICAM-1, neither bALP nor NO levels differed significantly between HD patients with or without CVD. As noted, previous studies have reported elevated serum NO levels in HD patients [39,40]. Nevertheless, at high concentrations, NO acts as a cytotoxic molecule and may contribute to dialysis-related complications due to its nature as a highly reactive free radical. Although elevated NO metabolites have been detected in HD patients, studies have reported contradictory findings regarding their role in atherosclerosis progression. Some investigations have shown a positive correlation between NO levels and carotid intima-media thickness or the presence of atherosclerotic plaques [41], whereas others have found no significant association with CVD [42].

Diabetes mellitus is the primary cause of ESKD and markedly elevates the risk of cardiovascular mortality in patients undergoing HD [38]. In our patient cohort, serum ICAM-1 levels did not differ between individuals with and without diabetes mellitus. Given that diabetes mellitus is a recognized risk factor for CVD in HD patients [38], the absence of a difference in ICAM-1 levels provides indirect evidence against its utility as a marker of CVD in this population. Serum bALP levels did not differ significantly between the two groups. Serum NO levels were elevated in HD patients with diabetes. Elevated NO levels are commonly observed in individuals with diabetes, potentially resulting from glucose-induced upregulation of NO synthase expression and activity [43]. It needs to be determined whether this represents a compensatory response to hyperglycemia-induced endothelial damage or a detrimental process. As previously noted, at high concentrations, NO functions as a cytotoxic molecule and may contribute to dialysis-related complications due to its reactivity as a free radical [41].

Low-grade inflammation is prevalent among HD patients and is associated with adverse clinical outcomes [24,44]. Using a CRP cut-off of 1 mg/dL to define inflammation, we found that inflammation in HD patients was associated with a significant increase in serum ICAM-1 levels, consistent with previous studies [16,25]. Serum bALP levels did not differ significantly between the two groups. Serum NO was significantly lower in HD patients with inflammation. Interestingly, experimental studies have demonstrated that CRP inhibits endothelial nitric oxide synthase activity directly [45]. Whether this mechanism contributes to inflammation-induced endothelial dysfunction and CVD in HD patients remains to be elucidated.

In HD patients, serum ICAM-1 showed a positive correlation with bALP, but no significant correlation with NO. Additionally, NO was not correlated with bALP. The observed correlation between serum ICAM-1 levels and bone turnover, as assessed by bALP, suggests that CKD-MBD may contribute to endothelial dysfunction. However, the absence of a correlation between serum bALP and NO, an indicator of endothelial dysfunction, argues against such an association. The observed negative correlation between serum NO and LDL cholesterol in our HD patients supports its utility as a marker of endothelial dysfunction, as LDL cholesterol suppresses endothelial NO production [46]. At the same time, statins or PCSK9 inhibitors reduce both LDL cholesterol and serum NO levels [47,48]. It should be noted that in our patients, LDL levels were relatively low because most were on statins. Therefore, LDL levels cannot be attributed to the protein-energy wasting syndrome, which is a significant factor of the reverse epidemiology that characterizes HD patients [49]. The lack of difference in serum NO between HD patients with and without CVD in our study may reflect confounding factors, such as reduced clearance or a compensatory response to uremia and associated hypertension [39,40].

Alternatively, the correlation between serum ICAM-1 levels and bALP may indicate that bone-derived ICAM-1 contributes to circulating levels. ICAM-1 is expressed on osteoblast surface, facilitating their interaction with osteoclast precursors and promoting osteoclast maturation. There are two types of osteoblasts: those that express ICAM-1 and promote the maturation of osteoclasts, and those that do not. Interestingly, the pro-inflammatory cytokine IL-1 enhances ICAM-1 expression in osteoblasts [50]. As a result, inflammatory conditions can increase both the expression and possible shedding of ICAM-1 by osteoblasts. Notably, in our study, patients with inflammation showed higher serum ICAM-1 levels. Mature osteoclasts also express ICAM-1 on their basolateral surface, which enhances their motility, rather than on the surface interacting with osteoblasts [51]. Parathyroid hormone enhances ICAM-1 expression in both osteoblasts and osteoclasts [52,53]. Interestingly, a study involving HD patients found that serum ICAM-1 levels were not associated with carotid intima-media thickness or CRP; however, they correlated with serum calcium and phosphate levels, which are related to bone metabolism [27]. Another study reported that cinacalcet, without vitamin D analog administration, in HD patients with secondary hyperparathyroidism reduced iPTH, calcium, and phosphate levels, decreased serum ICAM-1 levels, and had no effect on CRP [30]. The authors attributed the decrease in circulating ICAM-1 levels to the beneficial effects of cinacalcet-induced PTH lowering. However, the decreased expression and shedding of ICAM-1 by osteoblasts and osteoclasts due to lower PTH could also be responsible. Supporting the idea that high bone turnover contributes to circulating ICAM-1, forty patients with primary hyperparathyroidism exhibited elevated serum ICAM-1 levels [54]. The author hypothesizes that elevated PTH causes endothelial dysfunction; however, the potential for increased expression and shedding of ICAM-1 by osteoblasts and osteoclasts remains a possibility. The contribution of bone to circulating ICAM-1 levels could be better understood by studying a population with increased bone turnover and a healthy endothelium. Such a population is children. Indeed, healthy children exhibit higher bone turnover and serum ALP levels than adults, yet demonstrate normal endothelial function. Interestingly, in healthy children, serum levels of ICAM-1 are elevated and show a negative correlation with age [55,56]. Thus, serum ICAM-1 can originate from both the endothelium and bone. Accordingly, in the cohort of our patients, multiple linear regression including all the factors that were correlated with serum ICAM-1, i.e., bALP, ALP, duration of HD treatment, and inflammatory status (defined by a CRP cut-off of 1 mg/dL) demonstrated that bALP, ALP, and inflammatory status independently and positively predicted serum ICAM-1 levels.

Although this study is limited by its cross-sectional design, it can be regarded as an initial framework for subsequent prospective clinical research and experimental investigations. Another limitation of our study is that it included patients with a known history of CVD. This approach may have underestimated the true prevalence of CVD, as no specific diagnostic imaging tests were conducted during the study to identify clinically silent cases. However, HD patients are in the HD unit three times a week, so CVD symptoms are detected relatively early. Additionally, they undergo regular cardiological evaluations and transthoracic echocardiography. One more limitation is that the use of the phosphate binder sevelamer hydrochloride, the vitamin D analog paricalcitol, and the calcimimetic etelcalcetide was left to the discretion of the nephrologists, and these medications could potentially affect the evaluated outcomes. Nevertheless, their use was consistent with KDIGO recommendations for managing serum iPTH, calcium, and phosphorus levels, thus reflecting real-world clinical practice. In addition, bALP directly reflects bone turnover, regardless of whether therapeutic interventions are applied. Finally, despite the cell culture and clinical data, an in vivo model would clarify the extent of bone cells’ contribution to circulating ICAM-1 levels.

## 5. Conclusions

In conclusion, serum ICAM-1 levels do not differ between HD patients with or without a history of CVD, and ALP, bALP, and CRP are the main predictors of them.

Serum ICAM-1 is unlikely to be a reliable CVD marker in patients with HD. Inflammation and CKD-MBD confound the utility of serum ICAM-1 as a CVD marker in this population.

## Figures and Tables

**Table 1:** Patients’ characteristics.

	*N*	Mean	SD
Age (years)	142	66.36	11.74
Males/Females	102/40		
Duration on HD (months)	142	57.204	50.060
Residual diuresis (mL)	142	311.972	416.756
Hypertension (yes/no)	126/14		
Diabetes mellitus (yes/no)	50/92		
Cardiovascular disease (yes/no)	52/90		
Coronary heart disease (yes/no)	44/98		
Stroke (yes/no)	12/130		
Peripheral arterial disease (yes/no)	14/128		
Body mass index (Kg/m^2^)	142	27.018	5.422
White blood cell (c/μL)	142	7169.901	2519.412
Neutrophils (c/μL)	142	4816.183	1974.790
Lymphocytes (c/μL)	142	1699.514	583.352
Neutrophil-to-lymphocyte-ratio	142	3.099	1.445
Hemoglobin (g/dL)	142	11.742	0.827
Platelet (c/μL)	142	205.965	62.020
Urea (mg/dL)	142	128.301	27.975
Creatinine (mg/dL)	142	6.271	2.109
Urea reduction ratio (%)	142	67.478	7.249
Cholesterol (mg/dL)	142	132.894	41.066
Triglyceride (mg/dL)	142	133.317	72.737
LDL cholesterol (mg/dL)	142	76.942	36.490
Albumin (g/dL)	142	3.628	0.295
Alkaline phosphatase (U/L)	142	205.444	99.250
Ferritin (ng/mL)	142	173.920	178.414
Transferrin saturation (%)	142	18.496	11.502
Calcium (mg/dL)	142	9.169	0.476
Phosphorous (mg/dL)	142	5.283	0.986
Intact parathyroid hormone (pg/mL)	142	344.625	285.123
C-reactive protein (mg/dL)	142	1.441	0.842
C-reactive protein above 1 mg/dL (yes/no)	104/38		
Intercellular adhesion molecule-1 (ng/mL)	142	654.935	313.562
Bone alkaline phosphatase (ng/mL)	142	99.281	80.571
Nitric oxide (mg/dL)	132	0.056	0.035
Antihypertensive therapy (yes/no)	126/16		
Nitrate therapy (yes/no)	10/132		
Statin therapy (yes/no)	94/48		
Antiplatelet therapy (yes/no)	78/64		
Phosphate binders (yes/no)	126/16		
Vitamin D analogs (yes/no)	80/62		
Calcimimetics (yes/no)	40/102		

**Table 2:** Correlations among serum ICAM-1, bALP, and NO in HD patients.

		ICAM-1	bALP
**bALP**	Rho	0.204	
	*p*	0.016	
**NO**	Rho	−0.121	−0.061
	*p*	0.165	0.492

**Table 3:** Multiple Linear Regression Analysis of bALP, ALP, HD Duration, and CRP as Predictors of ICAM-1.

	β (Unstandardized)	SE	β (Standardized)	t	*p*
Constant	311.476	70.070		4.445	<0.001
bALP	0.844	0.315	0.216	2.674	0.008
ALP	0.815	0.251	0.259	3.246	0.001
Duration on HD	0.134	0.500	0.021	0.268	0.789
CRP > 1 mg/L	120.839	55.606	0.171	2.173	0.032
Model summary: R = 0.434, R^2^ = 0.189, Adjusted R^2^ = 0.165, *p* < 0.001					

## Data Availability

The original contributions presented in this study are included in the article and the Supplementary Material. Further inquiries may be directed to the corresponding author.

## Supplementary Materials

The following supporting information can be downloaded at: https://www.mdpi.com/article/10.3390/biom16010102/s1, Raw data.
