# Supplementary material for: Inflammation and Dysregulated Bone Turnover Confound Serum ICAM-1 as a Cardiovascular Marker in Hemodialysis
Source: Biomolecules. 2026 Jan 7;16(1):102. doi: 10.3390/biom16010102 (PMC12838869; doi:10.3390/biom16010102)
Supplement: Supplementary file 1 [file biomolecules-16-00102-s001.zip › biomolecules-4000327-supplementary.pdf]

Age	Sex	CVD	DM	Duration	Diuresis	BMI	WBC	Hb
66.00	0	1	1	33.00	1500.00	20.64	8500.00	12.30
78.00	1	0	0	14.00	0.00	19.04	5100.00	11.00
61.00	0	0	0	59.00	0.00	22.53	5600.00	12.50
70.00	1	0	0	79.00	0.00	29.49	7260.00	11.50
73.00	0	0	0	19.00	500.00	25.90	6500.00	11.60
53.00	0	0	0	31.00	0.00	27.03	6400.00	11.20
77.00	0	0	0	22.00	0.00	28.32	14530.00	10.80
43.00	0	0	0	121.00	0.00	41.21	6580.00	10.00
67.00	0	1	0	67.00	0.00	31.80	11950.00	12.40
82.00	1	1	0	75.00	0.00	23.52	3250.00	12.80
80.00	1	0	1	13.00	600.00	21.78	5040.00	11.90
55.00	1	0	0	15.00	700.00	25.61	8000.00	11.50
72.00	0	1	0	20.00	100.00	24.68	5700.00	10.40
73.00	1	0	1	115.00	0.00	37.50	8800.00	10.90
55.00	0	1	0	104.00	0.00	40.74	7030.00	12.50
48.00	0	1	1	31.00	1000.00	19.46	8100.00	11.30
68.00	1	0	0	51.00	500.00	25.78	7800.00	12.60
66.00	0	1	1	42.00	800.00	27.93	1058.00	10.90
60.00	0	0	0	53.00	0.00	23.46	5810.00	10.90
47.00	1	0	0	40.00	1000.00	22.59	5160.00	11.40
68.00	0	1	0	23.00	300.00	25.39	6840.00	10.90
62.00	0	1	0	162.00	0.00	26.30	8970.00	11.10
79.00	1	0	0	36.00	500.00	28.83	7750.00	12.20
77.00	1	0	0	31.00	300.00	26.63	5170.00	12.30
60.00	0	0	0	63.00	500.00	37.39	7340.00	11.40
65.00	0	1	1	34.00	1600.00	20.57	8550.00	12.40
77.00	1	0	0	15.00	0.00	19.01	5150.00	11.10
65.00	0	1	1	18.00	0.00	22.27	8480.00	12.80
64.00	0	0	0	62.00	700.00	22.84	5310.00	12.30
76.00	0	0	1	26.00	500.00	24.22	8440.00	11.10
64.00	0	0	0	68.00	0.00	24.33	3870.00	11.30
78.00	0	1	1	19.00	500.00	21.96	7720.00	11.80
59.00	0	0	0	261.00	0.00	22.41	8250.00	11.90
25.00	0	0	0	22.00	0.00	20.75	8330.00	12.70
58.00	1	0	0	78.00	0.00	22.77	5270.00	11.50
56.00	0	1	0	137.00	0.00	22.48	4570.00	10.20
76.00	0	0	0	13.00	500.00	29.71	4320.00	11.80
63.00	0	1	1	85.00	0.00	27.74	9900.00	12.00
76.00	1	1	1	29.00	0.00	21.33	10900.00	13.10
74.00	0	0	1	28.00	0.00	25.90	12120.00	12.40
80.00	0	0	0	2.00	1000.00	22.32	12600.00	11.00
80.00	0	1	0	19.00	0.00	24.09	7160.00	12.60
58.00	1	0	0	69.00	0.00	25.16	6350.00	11.10
78.00	0	1	0	75.00	0.00	28.03	4300.00	11.60
74.00	0	1	0	12.00	0.00	31.05	7080.00	12.40
79.00	0	0	0	109.00	0.00	25.43	8460.00	11.70
67.00	1	0	1	11.00	500.00	39.56	5700.00	11.50
80.00	0	1	1	26.00	0.00	25.33	4790.00	12.00
36.00	0	0	0	125.00	0.00	41.66	6180.00	12.90

73.00	0	1	1	7.00	1500.00	27.10	5370.00	9.70
79.00	0	0	0	5.00	0.00	26.95	11300.00	10.30
80.00	1	0	1	27.00	500.00	34.53	6500.00	11.60
65.00	0	0	1	45.00	0.00	23.78	9500.00	13.50
44.00	0	0	0	120.00	0.00	41.52	6530.00	9.90
68.00	0	1	0	66.00	0.00	32.00	11900.00	12.30
83.00	1	1	0	74.00	0.00	23.61	3200.00	12.70
81.00	1	0	1	12.00	500.00	21.85	4990.00	11.80
79.00	0	1	0	61.00	500.00	22.85	5210.00	12.50
72.00	0	0	1	21.00	0.00	24.00	3540.00	11.70
80.00	1	0	0	87.00	300.00	30.47	8740.00	12.10
68.00	1	0	1	7.00	500.00	29.91	10400.00	11.30
68.00	0	0	1	45.00	0.00	33.84	10610.00	12.30
43.00	0	0	0	58.00	0.00	29.01	4900.00	10.00
74.00	0	1	1	24.00	0.00	25.59	5700.00	11.90
75.00	0	1	1	47.00	100.00	23.81	7200.00	12.10
66.00	1	0	1	12.00	600.00	39.26	5750.00	11.60
79.00	0	1	1	27.00	0.00	25.21	4840.00	12.10
60.00	0	0	0	60.00	0.00	22.44	5650.00	12.60
69.00	1	0	0	80.00	0.00	29.32	7310.00	11.60
66.00	0	0	0	161.00	1100.00	23.77	5460.00	11.60
79.00	0	1	1	25.00	900.00	28.73	13950.00	12.20
50.00	0	1	1	21.00	1100.00	29.71	5200.00	10.60
52.00	0	0	0	9.00	800.00	41.36	7690.00	10.50
71.00	0	1	1	56.00	600.00	24.57	9970.00	11.10
59.00	0	0	0	95.00	0.00	30.12	5660.00	12.70
64.00	0	0	0	130.00	0.00	24.30	5890.00	12.70
72.00	1	0	0	18.00	1100.00	19.74	6210.00	12.60
59.00	1	0	0	18.00	600.00	23.95	6520.00	12.50
68.00	0	0	0	159.00	0.00	32.07	7680.00	11.70
54.00	1	0	1	158.00	0.00	29.03	5240.00	11.40
75.00	0	1	0	145.00	0.00	26.79	5310.00	13.10
54.00	0	1	0	105.00	0.00	40.44	7080.00	12.60
47.00	0	1	1	32.00	1100.00	19.40	8150.00	11.40
67.00	1	0	0	52.00	600.00	25.65	7850.00	12.70
65.00	0	1	1	43.00	900.00	27.78	1108.00	11.00
59.00	0	0	0	54.00	0.00	23.35	5860.00	11.00
46.00	1	0	0	41.00	1100.00	22.50	5210.00	11.50
67.00	0	1	0	24.00	300.00	25.27	6890.00	11.00
61.00	0	1	0	163.00	0.00	26.16	9020.00	11.20
78.00	1	0	0	37.00	600.00	28.67	7800.00	12.30
76.00	1	0	0	32.00	300.00	26.49	5220.00	12.40
59.00	0	0	0	64.00	600.00	37.13	7390.00	11.50
52.00	0	0	0	32.00	0.00	26.88	6450.00	11.30
76.00	0	0	0	23.00	0.00	28.16	14580.00	10.90
64.00	0	1	1	19.00	0.00	22.18	8530.00	12.90
63.00	0	0	0	63.00	800.00	22.74	5360.00	12.40
75.00	0	0	1	27.00	600.00	24.11	8490.00	11.20
63.00	0	0	0	69.00	0.00	24.21	3920.00	11.40
77.00	0	1	1	20.00	600.00	21.88	7770.00	11.90

58.00	0	0	0	262.00	0.00	22.32	8300.00	12.00
24.00	0	0	0	23.00	0.00	20.69	8380.00	12.80
57.00	1	0	0	79.00	0.00	22.68	5320.00	11.60
55.00	0	1	0	138.00	0.00	22.39	4620.00	10.30
75.00	0	0	0	14.00	600.00	29.54	4370.00	11.90
62.00	0	1	1	86.00	0.00	27.59	9950.00	12.10
75.00	1	1	1	30.00	0.00	21.27	10950.00	13.20
73.00	0	0	1	29.00	0.00	25.77	12170.00	12.50
79.00	0	0	0	3.00	1100.00	22.23	12650.00	11.10
79.00	0	1	0	20.00	0.00	23.98	7210.00	12.70
57.00	1	0	0	70.00	0.00	25.03	6400.00	11.20
77.00	0	1	0	76.00	0.00	27.87	4350.00	11.70
73.00	0	1	0	13.00	0.00	30.86	7130.00	12.50
78.00	0	0	0	110.00	0.00	25.31	8510.00	11.80
79.00	1	0	1	28.00	600.00	34.29	6550.00	11.70
35.00	0	0	0	126.00	0.00	41.35	6230.00	13.00
72.00	0	1	1	8.00	1600.00	26.96	5420.00	9.80
78.00	0	0	0	6.00	0.00	26.81	11350.00	10.40
72.00	0	0	0	20.00	600.00	25.77	6550.00	11.70
64.00	0	0	1	46.00	0.00	23.68	9550.00	13.60
78.00	0	1	0	62.00	600.00	22.76	5260.00	12.60
71.00	0	0	1	22.00	0.00	23.89	3590.00	11.80
79.00	1	0	0	88.00	300.00	30.28	8790.00	12.20
67.00	1	0	1	8.00	600.00	29.73	10450.00	11.40
67.00	0	0	1	46.00	0.00	33.61	10660.00	12.40
54.00	1	0	0	16.00	800.00	25.48	8050.00	11.60
71.00	0	1	0	21.00	200.00	24.56	5750.00	10.50
72.00	1	0	1	116.00	0.00	37.23	8850.00	11.00
42.00	0	0	0	59.00	0.00	28.85	4950.00	10.10
73.00	0	1	1	25.00	0.00	25.46	5750.00	12.00
74.00	0	1	1	48.00	0.00	23.70	7250.00	12.20
67.00	0	0	0	160.00	1000.00	23.88	5410.00	11.50
80.00	0	1	1	24.00	800.00	28.89	13900.00	12.10
51.00	0	1	1	20.00	1000.00	29.89	5150.00	10.50
53.00	0	0	0	8.00	700.00	41.67	7640.00	10.40
72.00	0	1	1	55.00	500.00	24.69	9920.00	11.00
60.00	0	0	0	94.00	0.00	30.30	5610.00	12.60
65.00	0	0	0	129.00	0.00	24.41	5840.00	12.60
73.00	1	0	0	17.00	1000.00	19.78	6160.00	12.50
60.00	1	0	0	17.00	500.00	24.06	6470.00	12.40
69.00	0	0	0	158.00	0.00	32.27	7630.00	11.60
55.00	1	0	1	157.00	0.00	29.20	5190.00	11.30
76.00	0	1	0	144.00	0.00	26.94	5260.00	13.00

PLT	Urea	URR	Creat	Chol	TRG	cLDL	ALP	Ferritin
156.00	81.00	65.43	3.60	81.00	108.00	36.06	218.00	118.00
155.00	131.00	76.34	4.30	140.00	162.00	76.74	215.00	176.80
154.00	113.00	65.49	5.00	151.00	108.00	99.06	141.00	203.00
223.00	120.00	71.67	6.00	203.00	63.00	156.21	231.00	54.40
83.00	167.00	71.86	7.10	144.00	144.00	84.48	166.00	67.70
158.00	118.00	68.64	8.60	170.00	97.00	118.69	157.00	11.20
374.00	108.00	64.81	2.10	166.00	109.00	112.33	157.00	45.00
157.00	132.00	49.24	10.20	113.00	221.00	38.87	151.00	110.60
272.00	172.00	72.67	8.90	194.00	365.00	78.65	216.00	313.60
122.00	113.00	67.26	6.40	172.00	92.00	121.64	191.00	460.60
200.00	151.00	76.16	6.20	123.00	138.00	66.96	196.00	149.00
307.00	73.00	63.01	7.80	160.00	207.00	84.39	144.00	190.00
143.00	99.00	68.69	9.70	107.00	137.00	52.79	95.00	180.00
244.00	136.00	63.24	8.40	112.00	194.00	44.18	75.00	290.00
202.00	170.00	74.71	9.20	143.00	202.00	70.24	224.00	53.30
237.00	122.00	69.67	5.90	258.00	213.00	171.21	295.00	87.00
180.00	96.00	58.33	3.80	165.00	109.00	111.43	203.00	556.00
239.00	120.00	63.33	4.60	61.00	106.00	18.52	315.00	162.80
196.00	160.00	74.38	7.70	158.00	75.00	112.95	241.00	61.30
169.00	114.00	69.30	5.40	152.00	138.00	93.06	217.00	80.70
211.00	181.00	74.59	6.40	120.00	80.00	77.60	130.00	258.90
225.00	121.00	69.42	9.20	150.00	244.00	66.88	170.00	319.40
182.00	94.00	64.89	5.00	172.00	157.00	106.69	221.00	709.70
236.00	130.00	73.85	3.60	119.00	191.00	51.17	200.00	536.80
175.00	133.00	58.65	6.80	146.00	60.00	105.60	113.00	213.40
159.00	83.00	65.06	3.70	80.00	109.00	34.93	219.00	119.60
158.00	133.00	75.94	4.40	139.00	163.00	75.61	216.00	178.40
242.00	177.00	73.45	6.40	145.00	84.00	99.18	286.00	106.80
202.00	160.00	76.88	6.70	192.00	200.00	114.80	186.00	46.20
142.00	165.00	77.58	5.80	78.00	124.00	29.68	304.00	228.40
194.00	112.00	54.46	6.40	131.00	48.00	94.86	159.00	141.10
393.00	105.00	58.10	2.90	124.00	191.00	55.67	291.00	44.00
174.00	138.00	65.22	5.60	73.00	59.00	40.13	200.00	122.00
288.00	120.00	72.50	10.40	140.00	70.00	97.90	208.00	83.50
247.00	126.00	79.37	7.10	152.00	84.00	105.48	173.00	107.00
142.00	99.00	67.68	6.00	93.00	46.00	61.12	166.00	21.70
230.00	144.00	65.97	4.80	75.00	33.00	47.91	147.00	15.80
359.00	134.00	63.43	7.70	161.00	410.00	38.60	166.00	30.90
236.00	90.00	71.11	4.30	127.00	129.00	72.63	223.00	103.00
248.00	96.00	84.38	5.60	94.00	280.00	8.20	380.00	940.70
157.00	125.00	70.40	5.00	139.00	90.00	92.40	152.00	13.70
204.00	109.00	60.55	5.10	162.00	104.00	109.88	197.00	120.00
199.00	142.00	79.58	6.10	167.00	151.00	103.57	220.00	16.20
95.00	94.00	65.96	4.60	151.00	72.00	107.34	190.00	59.00
193.00	140.00	71.43	6.00	87.00	108.00	41.46	179.00	74.20
297.00	111.00	60.36	5.40	111.00	61.00	73.87	172.00	373.10
185.00	105.00	69.52	5.40	147.00	102.00	96.84	124.00	29.80
160.00	86.00	45.35	2.90	137.00	177.00	70.59	141.00	17.90
204.00	146.00	58.90	9.00	95.00	138.00	41.76	227.00	34.00

234.00	154.00	67.53	6.50	87.00	113.00	40.31	187.00	15.00
119.00	118.00	70.34	2.20	183.00	65.00	137.75	208.00	498.00
286.00	70.00	68.57	4.60	97.00	110.00	50.00	340.00	246.90
250.00	190.00	74.21	6.00	123.00	100.00	75.70	194.00	431.00
154.00	130.00	49.23	10.10	114.00	220.00	40.00	150.00	109.00
269.00	170.00	72.94	8.80	195.00	364.00	79.78	215.00	312.00
119.00	111.00	67.57	6.30	173.00	91.00	122.77	190.00	459.00
197.00	149.00	76.51	6.10	124.00	137.00	68.09	195.00	147.40
126.00	124.00	69.35	6.00	121.00	46.00	86.32	143.00	26.00
187.00	66.00	66.67	4.40	126.00	104.00	77.48	217.00	48.40
155.00	118.00	74.58	5.40	131.00	98.00	83.36	178.00	434.60
159.00	174.00	66.67	5.00	138.00	199.00	66.43	174.00	89.70
217.00	130.00	63.85	7.50	137.00	74.00	94.28	153.00	62.90
237.00	126.00	73.02	14.20	125.00	110.00	75.20	49.00	158.00
210.00	147.00	65.31	3.70	143.00	201.00	70.47	73.00	153.00
212.00	181.00	71.27	9.80	101.00	147.00	45.09	109.00	146.00
188.00	107.00	69.16	5.50	146.00	103.00	95.71	125.00	31.40
163.00	88.00	45.45	3.00	136.00	178.00	69.46	142.00	19.50
157.00	115.00	65.22	5.10	150.00	109.00	97.93	142.00	204.60
226.00	122.00	71.31	6.10	202.00	64.00	155.08	232.00	56.00
165.00	172.00	62.79	7.60	122.00	82.00	78.94	544.00	16.70
148.00	119.00	52.94	3.20	87.00	119.00	38.93	283.00	58.90
299.00	148.00	63.51	7.40	85.00	178.00	23.56	147.00	377.60
168.00	142.00	61.27	8.00	153.00	165.00	87.75	132.00	398.40
284.00	146.00	70.55	7.10	91.00	87.00	49.89	198.00	68.30
120.00	151.00	76.82	8.10	89.00	168.00	29.46	299.00	87.10
172.00	120.00	67.50	6.90	101.00	36.00	70.62	161.00	71.80
170.00	111.00	57.66	4.60	286.00	108.00	220.56	737.00	189.60
292.00	123.00	65.85	6.80	110.00	153.00	51.81	175.00	134.60
169.00	123.00	52.85	6.50	79.00	79.00	40.93	362.00	134.60
153.00	99.00	72.73	4.50	198.00	327.00	90.99	156.00	58.20
242.00	140.00	73.57	6.20	81.00	96.00	38.82	175.00	273.60
205.00	172.00	74.42	9.30	142.00	203.00	69.11	225.00	54.90
240.00	124.00	69.35	6.00	257.00	214.00	170.08	296.00	88.60
183.00	98.00	58.16	3.90	164.00	110.00	110.30	204.00	557.60
242.00	122.00	63.11	4.70	60.00	107.00	17.39	316.00	164.40
199.00	162.00	74.07	7.80	157.00	76.00	111.82	242.00	62.90
172.00	116.00	68.97	5.50	151.00	139.00	91.93	218.00	82.30
214.00	183.00	74.32	6.50	119.00	81.00	76.47	131.00	260.50
228.00	123.00	69.11	9.30	149.00	245.00	65.75	171.00	321.00
185.00	96.00	64.58	5.10	171.00	158.00	105.56	222.00	711.30
239.00	132.00	73.48	3.70	118.00	192.00	50.04	201.00	538.40
178.00	135.00	58.52	6.90	145.00	61.00	104.47	114.00	215.00
161.00	120.00	68.33	8.70	169.00	98.00	117.56	158.00	12.80
377.00	110.00	64.55	2.20	165.00	110.00	111.20	158.00	46.60
245.00	179.00	73.18	6.50	144.00	85.00	98.05	287.00	108.40
205.00	162.00	76.54	6.80	191.00	201.00	113.67	187.00	47.80
145.00	167.00	77.25	5.90	77.00	125.00	28.55	305.00	230.00
197.00	114.00	54.39	6.50	130.00	49.00	93.73	160.00	142.70
396.00	107.00	57.94	3.00	123.00	192.00	54.54	292.00	45.60

177.00	140.00	65.00	5.70	72.00	60.00	39.00	201.00	123.60
291.00	122.00	72.13	10.50	139.00	71.00	96.77	209.00	85.10
250.00	128.00	78.91	7.20	151.00	85.00	104.35	174.00	108.60
145.00	101.00	67.33	6.10	92.00	47.00	59.99	167.00	23.30
233.00	146.00	65.75	4.90	74.00	34.00	46.78	148.00	17.40
362.00	136.00	63.24	7.80	160.00	411.00	37.47	167.00	32.50
239.00	92.00	70.65	4.40	126.00	130.00	71.50	224.00	104.60
251.00	98.00	83.67	5.70	93.00	281.00	7.07	381.00	942.30
160.00	127.00	70.08	5.10	138.00	91.00	91.27	153.00	15.30
207.00	111.00	60.36	5.20	161.00	105.00	108.75	198.00	121.60
202.00	144.00	79.17	6.20	166.00	152.00	102.44	221.00	17.80
98.00	96.00	65.63	4.70	150.00	73.00	106.21	191.00	60.60
196.00	142.00	71.13	6.10	86.00	109.00	40.33	180.00	75.80
300.00	113.00	60.18	5.50	110.00	62.00	72.74	173.00	374.70
289.00	72.00	68.06	4.70	96.00	111.00	48.87	341.00	248.50
207.00	148.00	58.78	9.10	94.00	139.00	40.63	228.00	35.60
237.00	156.00	67.31	6.60	86.00	114.00	39.18	188.00	16.60
122.00	120.00	70.00	2.30	182.00	66.00	136.62	209.00	499.60
86.00	169.00	71.60	7.20	143.00	145.00	83.35	167.00	69.30
253.00	192.00	73.96	6.10	122.00	101.00	74.57	195.00	432.60
129.00	126.00	69.05	6.10	120.00	47.00	85.19	144.00	27.60
190.00	68.00	66.18	4.50	125.00	105.00	76.35	218.00	50.00
158.00	120.00	74.17	5.50	130.00	99.00	82.23	179.00	436.20
162.00	176.00	66.48	5.10	137.00	200.00	65.30	175.00	91.30
220.00	132.00	63.64	7.60	136.00	75.00	93.15	154.00	64.50
310.00	75.00	62.67	7.90	159.00	208.00	83.26	145.00	191.60
146.00	101.00	68.32	9.80	106.00	138.00	51.66	96.00	181.60
247.00	138.00	63.04	8.50	111.00	195.00	43.05	76.00	291.60
240.00	128.00	72.66	14.30	124.00	111.00	74.07	50.00	159.60
213.00	149.00	65.10	3.80	142.00	202.00	69.34	74.00	154.60
215.00	183.00	71.04	9.90	100.00	148.00	43.96	110.00	147.60
162.00	170.00	62.94	7.50	123.00	81.00	80.07	543.00	15.10
145.00	117.00	52.99	3.10	88.00	118.00	40.06	282.00	57.30
296.00	146.00	63.70	7.30	86.00	177.00	24.69	146.00	376.00
165.00	140.00	61.43	7.90	154.00	164.00	88.88	131.00	396.80
281.00	144.00	70.83	7.00	92.00	86.00	51.02	197.00	66.70
117.00	149.00	77.18	8.00	90.00	167.00	30.59	298.00	85.50
169.00	118.00	67.80	6.80	102.00	35.00	71.75	160.00	70.20
167.00	109.00	57.80	4.50	287.00	107.00	221.69	736.00	188.00
289.00	121.00	66.12	6.70	111.00	152.00	52.94	174.00	133.00
166.00	121.00	52.89	6.40	80.00	78.00	42.06	361.00	133.00
150.00	97.00	73.20	4.40	199.00	326.00	92.12	155.00	56.60
239.00	138.00	73.91	6.10	82.00	95.00	39.95	174.00	272.00

TSAT	Ca	P	Alb	CRP	CRP1	PTH	BALPp	ICAMp
17.04	8.80	5.10	3.70	1.01	1	88.2	37.908	684.952
11.38	9.40	4.20	3.70	0.53	0	185.4	55.779	63.382
20.96	9.20	5.20	3.60	0.87	0	172.0	195.897	421.730
10.33	8.80	4.80	3.50	2.02	1	248.2	102.586	1332.152
21.47	8.80	6.60	3.70	2.44	1	166.0	94.168	369.446
10.48	8.60	5.50	3.70	2.00	1	454.7	79.228	605.648
5.76	8.90	3.80	3.60	2.14	1	95.1	23.731	448.426
40.79	8.70	8.10	3.70	1.54	1	803.9	60.823	657.036
12.98	9.50	4.90	3.60	0.79	0	181.6	32.362	586.802
28.27	9.10	4.30	3.30	1.03	0	64.5	64.061	285.000
29.04	9.10	6.30	3.20	2.02	1	267.9	125.472	281.316
20.40	9.48	6.00	4.20	0.34	0	238.0	17.084	723.480
23.16	9.10	3.80	4.40	0.47	0	199.0	54.653	1040.342
17.98	9.10	5.50	4.40	0.46	0	490.0	133.094	662.850
25.20	10.10	7.10	4.00	1.71	1	254.1	143.642	1223.602
17.43	8.90	6.50	3.70	0.49	0	391.6	117.144	528.896
32.98	9.10	5.00	3.30	1.47	1	339.9	77.693	1232.754
10.85	8.40	4.20	3.50	1.80	1	803.8	141.409	868.776
14.66	9.00	5.80	3.70	1.11	1	369.5	257.025	868.872
18.54	10.20	5.00	3.70	1.47	1	834.8	358.750	1123.600
23.19	10.00	6.10	3.40	2.11	1	255.4	11.230	946.398
15.43	10.10	5.00	3.90	1.77	1	586.4	238.321	489.554
16.21	9.50	4.80	3.60	1.40	1	502.5	115.499	762.222
19.24	9.20	4.90	3.80	1.06	1	365.1	78.634	837.384
16.75	8.80	5.90	3.20	1.66	1	471.8	181.510	284.060
17.22	8.90	5.20	3.60	1.04	1	89.4	38.577	671.560
11.60	9.50	4.30	3.60	0.56	0	186.6	56.182	94.996
10.35	9.10	6.00	3.80	1.96	1	205.6	97.504	1201.110
15.76	9.20	5.40	3.90	1.06	1	300.2	32.802	353.172
41.32	8.70	4.70	3.30	2.33	1	575.6	25.726	519.936
16.70	8.90	5.10	4.10	1.03	1	90.6	59.488	921.138
6.69	8.60	5.20	3.30	1.17	1	300.1	54.499	724.962
8.52	9.20	4.20	3.70	2.63	1	124.7	72.443	517.092
11.94	9.50	8.50	3.80	2.07	1	497.3	321.582	663.372
16.60	9.00	4.40	3.70	1.12	1	302.9	118.604	585.942
5.63	8.40	4.30	3.70	1.39	1	200.5	60.027	569.622
5.45	9.20	5.50	3.50	1.77	1	287.4	71.230	181.652
13.39	9.00	6.70	3.50	2.12	1	260.8	36.450	237.580
17.29	8.70	4.50	3.60	6.36	1	153.6	44.281	336.360
26.84	8.80	6.10	3.50	1.03	1	310.1	24.488	437.932
9.92	9.50	5.50	3.70	1.77	1	61.4	5.261	793.518
33.12	9.10	4.50	3.50	0.81	0	1719.5	115.113	559.686
6.52	9.90	6.00	4.00	1.02	1	120.2	124.550	1199.534
13.63	9.10	5.10	3.50	1.04	1	230.6	25.053	641.274
14.72	8.70	4.60	3.60	1.11	1	327.9	100.201	353.696
16.49	9.10	5.60	2.90	2.20	1	207.1	97.653	664.520
11.19	9.50	5.40	3.60	1.67	1	72.0	21.279	457.672
11.26	8.60	4.50	3.70	0.99	0	290.2	87.431	710.406
6.26	9.10	6.10	3.40	1.63	1	496.0	368.486	1417.678

6.96	8.20	5.10	3.50	2.57	1	262.8	156.125	757.504
27.35	9.10	5.40	3.70	1.70	1	120.1		405.896
28.26	8.60	3.80	2.70	2.90	1	493.5	89.321	696.588
35.02	9.80	5.20	3.80	0.88	0	581.0	218.553	920.938
40.84	8.60	8.00	3.80	1.51	1	802.7	60.906	690.818
12.77	9.40	4.80	3.70	0.76	0	180.4	31.834	607.980
28.18	9.00	4.20	3.40	1.00	0	63.3	64.274	331.310
28.97	9.00	6.20	3.30	1.99	1	266.7	126.699	294.186
12.14	8.60	5.90	3.60	1.03	1	63.0	9.512	1013.058
19.64	9.00	3.80	3.60	0.77	0	131.0	17.408	622.366
14.27	9.50	3.70	3.60	1.15	1	73.1	45.529	508.680
19.99	8.50	6.10	3.40	1.61	1	202.5	89.870	598.644
16.64	9.10	6.20	4.10	1.05	1	154.4	40.201	794.412
86.58	8.50	4.80	3.80	0.70	0	217.0	58.142	388.326
23.63	9.20	5.00	4.20	0.35	0	82.0	19.722	372.972
21.39	8.60	6.60	3.80	0.16	0	180.0	46.797	576.340
11.42	9.60	5.50	3.50	1.70	1	73.2	21.439	451.172
11.46	8.70	4.60	3.60	0.98	0	291.4	87.387	716.448
21.14	9.30	5.30	3.50	0.90	0	173.2	193.290	419.810
10.57	8.90	4.90	3.40	2.05	1	249.4	103.351	1312.528
10.17	8.80	4.50	4.00	0.72	0	1141.2	189.093	309.914
8.45	9.10	4.50	3.70	1.44	1	324.7	62.774	279.036
19.49	10.40	6.80	4.10	0.93	0	780.0	199.207	318.276
27.65	9.10	4.10	3.50	1.04	1	281.1	34.200	204.648
12.60	10.10	5.70	3.50	1.27	1	1063.2	91.544	614.890
19.09	9.10	5.50	3.80	1.58	1	422.2	204.358	725.186
15.44	10.10	4.10	3.70	1.14	1	318.0	140.122	729.628
26.96	9.40	5.20	3.60	1.91	1	240.8	123.541	1749.540
22.59	9.20	4.00	3.70	0.67	0	239.8	19.891	607.880
13.95	9.20	4.10	3.60	1.56	1	150.9	110.768	796.540
8.12	9.00	4.80	3.40	0.98	0	190.4	11.678	655.248
13.37	10.10	6.00	3.70	1.63	1	768.2	207.732	924.952
25.28	10.20	7.20	3.90	1.74	1	255.3	145.553	1186.024
17.63	9.00	6.60	3.60	0.52	0	392.8	118.971	530.760
33.01	9.20	5.10	3.20	1.50	1	341.1	79.508	1210.956
11.11	8.50	4.30	3.40	1.83	1	805.0	142.462	856.226
14.86	9.10	5.90	3.60	1.14	1	370.7	253.778	843.268
18.71	10.30	5.10	3.60	1.50	1	836.0	353.545	1105.996
23.34	10.10	6.20	3.30	2.14	1	256.6	11.412	925.570
15.66	10.20	5.10	3.80	1.80	1	587.6	234.051	501.544
16.39	9.60	4.90	3.50	1.43	1	503.7	115.066	746.694
19.40	9.30	5.00	3.70	1.09	1	366.3	79.063	813.360
17.04	8.90	6.00	3.10	1.69	1	473.0	180.984	305.904
10.65	8.70	5.60	3.60	2.03	1	455.9	79.947	611.524
6.01	9.00	3.90	3.50	2.17	1	96.3	24.105	450.716
10.57	9.20	6.10	3.70	1.99	1	206.8	99.376	1191.026
15.93	9.30	5.50	3.80	1.09	1	301.4	34.429	371.146
41.24	8.80	4.80	3.20	2.36	1	576.8	26.302	513.518
16.90	9.00	5.20	4.00	1.06	1	91.8	59.902	896.162
6.94	8.70	5.30	3.20	1.20	1	301.3	55.571	745.744

8.73	9.30	4.30	3.60	2.66	1	125.9	73.020	543.280
12.17	9.60	8.60	3.70	2.10	1	498.5	319.711	675.544
16.78	9.10	4.50	3.60	1.15	1	304.1	116.608	600.302
5.86	8.50	4.40	3.60	1.42	1	201.7	59.857	566.574
5.71	9.30	5.60	3.40	1.80	1	288.6	71.477	204.122
13.53	9.10	6.80	3.40	2.15	1	262.0	36.770	219.910
17.58	8.80	4.60	3.50	6.39	1	154.8	44.918	349.212
26.96	8.90	6.20	3.40	1.06	1	311.3	25.576	436.754
10.18	9.60	5.60	3.60	1.80	1	62.6	5.125	782.696
33.15	9.20	4.60	3.40	0.84	0	1720.7	113.937	541.814
6.73	10.00	6.10	3.90	1.05	1	121.4	124.046	1137.778
13.82	9.20	5.20	3.40	1.07	1	231.8	25.280	640.314
14.95	8.80	4.70	3.50	1.14	1	329.1	99.486	357.666
16.78	9.20	5.70	2.80	2.23	1	208.3	96.036	602.856
28.36	8.70	3.90	2.60	2.93	1	494.7	89.032	699.048
6.50	9.20	6.20	3.30	1.66	1	497.2	364.667	1372.600
7.22	8.30	5.20	3.40	2.60	1	264.0	153.975	704.244
27.43	9.20	5.50	3.60	1.73	1	121.3		422.808
21.59	8.90	6.70	3.60	2.47	1	167.2	93.555	341.340
35.02	9.90	5.30	3.70	0.91	0	582.2	216.243	659.474
12.34	8.70	6.00	3.50	1.06	1	64.2	10.041	930.606
19.78	9.10	3.90	3.50	0.80	0	132.2	17.906	602.778
14.52	9.60	3.80	3.50	1.18	1	74.3	45.883	399.264
20.15	8.60	6.20	3.30	1.64	1	203.7	90.481	485.558
16.77	9.20	6.30	4.00	1.08	1	155.6	40.914	686.956
20.53	9.58	6.10	4.10	0.37	0	239.2	17.229	722.192
23.28	9.20	3.90	4.30	0.50	0	200.2	54.985	1039.018
18.15	9.20	5.60	4.30	0.49	0	491.2	130.573	617.340
86.09	8.60	4.90	3.70	0.73	0	218.2	58.612	366.978
23.74	9.30	5.10	4.10	0.38	0	83.2	19.857	342.594
21.54	8.70	6.70	3.70	0.19	0	181.2	46.861	556.162
9.97	8.70	4.40	4.10	0.69	0	1140.0	192.308	301.666
8.22	9.00	4.40	3.80	1.41	1	323.5	62.876	270.854
19.34	10.30	6.70	4.20	0.90	0	778.8	200.275	288.170
27.55	9.00	4.00	3.60	1.01	1	279.9	34.243	197.026
12.40	10.00	5.60	3.60	1.24	1	1062.0	92.501	683.568
18.91	9.00	5.40	3.90	1.55	1	421.0	209.660	761.670
15.24	10.00	4.00	3.80	1.11	1	316.8	141.702	745.760
26.88	9.30	5.10	3.70	1.88	1	239.6	126.057	1709.982
22.46	9.10	3.90	3.80	0.64	0	238.6	20.187	627.826
13.71	9.10	4.00	3.70	1.53	1	149.7	111.834	808.032
7.93	8.90	4.70	3.50	0.97	0	189.2	11.761	673.282
13.11	10.00	5.90	3.80	1.60	1	767.0	207.433	980.162

NOp	BALPh	ICAMh	NOh
0.0592	3.423	338.572	0.030
0.0641	3.823	276.589	0.037
0.1136	133.659	591.170	0.034
0.0159	40.379	137.557	0.024
0.0505	23.609	221.290	0.039
0.0656	3.426	400.061	0.046
0.0404	129.400	563.541	0.035
0.0954	40.556	149.649	0.024
0.0518	23.887	204.077	0.039
0.0397	5.209	838.966	0.023
0.0913	5.262	892.529	0.022
0.0660	4.468		0.046
	4.204	255.927	0.038
0.1344	8.379	777.001	0.035
0.0329	9.193	246.046	0.077
0.0263	3.355	370.869	0.029
0.0283	14.507	194.633	0.044
0.0257	3.212	471.991	0.046
0.0798	15.038	188.834	0.045
0.0347	7.734	798.327	0.035
0.0356	3.204		0.045
0.0308	8.592	264.627	0.074
0.0538	4.112	201.918	0.057
0.0301	3.809	624.160	0.038
0.0745	3.524	239.254	0.057
0.0600	3.582	643.847	0.038
0.0669			
0.0510			
0.0430			
0.0546			
0.0257			
0.0777			
0.0697			
0.0131			
0.0184			
0.0287			
0.0457			
0.0487			
0.1630			
0.0907			
0.0585			
0.0355			
0.0740			
0.0225			
0.0233			
0.1019			
0.2209			

0.0508
0.0443
0.0523
0.0884
0.0942
0.0514
0.0388
0.0903
0.0340
0.0873
0.0505

0.0505
0.0230
0.0362
0.0505
0.0239
0.1046
0.1169
0.0165
0.0226

0.0974
0.0569
0.0618
0.0320
0.0359
0.0160
0.0214
0.0596
0.0940
0.0519
0.0337
0.0270
0.0293
0.0268
0.0252
0.0356
0.0493
0.0312
0.0552
0.0302
0.0768
0.0600
0.0408
0.0524
0.0436
0.0552
0.0262
0.0758

0.0592
0.0152
0.0190
0.0295
0.0465
0.0493
0.1298
0.0923
0.0542
0.0362
0.0692

0.0229

0.0533
0.2241
0.0516
0.0451
0.0511
0.0892
0.0347
0.0886
0.0493

0.0515
0.0668

0.1362
0.0236
0.0367
0.0905
0.0218

0.0965
0.0556
0.0617
0.0310
0.0344
0.0153
0.0206
0.0678
0.0922
0.0511
